# Gliomas grading prediction using multicenter 3T Magnetic Resonance Spectroscopy

**DOI:** 10.1186/s12880-026-02640-5

**Published:** 2026-07-31

**Authors:** Di Guo, Kaiwei Xie, Jiacheng Lin, Jianshu Chen, Jialue Zhang, Meijin Lin, Xiaobo Qu, Wanjun Hu, Jianhua Wang, Dongyun Liang

**Affiliations:** 1 School of Computer and Information Engineering, Fujian Engineering Research Center for Medical Data Mining and Application, Xiamen University of Technology, Xiamen, 361024 China; 2 Department of Electronic Science, Fujian Provincial Key Laboratory of Plasma and Magnetic Resonance, School of Electronic Science and Engineering, Xiamen University, Xiamen, 361005 China; 3 Department of Applied Marine Physics and Engineering, Fujian Provincial Key Laboratory of Plasma and Magnetic Resonance, Xiamen University, Xiamen, 361005 China; 4 Department of Nuclear Magnetic Resonance, The Second Hospital & Clinical Medical School, Gansu Province Clinical Research Center for Functional and Molecular Imaging, Lanzhou University, Lanzhou, 730030 China; 5 Department of Radiology, The First Affiliated Hospital of Xiamen University, 55 Zhenhai Road, Siming District, Xiamen, 361000 China; 6 Department of Radiology, Zhongshan Hospital (Xiamen), Xiamen Municipal Clinical Research Center for Medical Imaging, Fujian Province Key Clinical Specialty for Medical Imaging, Xiamen Key Laboratory of Clinical Transformation of Imaging Big Data and Artificial Intelligence, Fudan University, Xiamen, 361015 China

**Keywords:** Magnetic Resonance Spectroscopy, Glioma grading, Metabolites, Multicenter study

## Abstract

**Background:**

Magnetic Resonance Spectroscopy (MRS) is a non-invasive imaging technique that detects various metabolites in brain tissues, making it essential for the grading of brain gliomas. MRS measures key metabolites such as N-acetyl-aspartate (NAA), choline (Cho), and creatine (Cr), whose ratio changes provide valuable information for glioma grading, guiding diagnosis, treatment, and prognosis. Current studies are largely single-center, with grading criteria differing across hospitals. This work aims to identify reliable and consistent metabolite ratios for glioma grading by jointly analyzing multicenter 3T single-voxel MRS of brain gliomas. The grading accuracy of these metabolite ratios is evaluated to establish a more standardized diagnostic basis.

**Materials and methods:**

We retrospectively included the MRS data from 60 glioma patients from 3 hospitals. MRS results included 4 metabolite ratios: NAA/Cr, Cho/NAA, Cho/Cr, and NAA/(Cho + Cr). The Shapiro-Wilk test was employed to ascertain the normality of the data, and the differences were calculated using either an independent *t*-test or a Mann-Whitney U-test. The classification performance was evaluated using ROC curve and accuracy analysis.

**Results:**

Across multicenter data, statistically significant differences were found in Cho/NAA and Cho/Cr ratios between low- and high-grade gliomas (*P* < 0.05). ROC analysis showed AUCs of 0.73 for Cho/NAA and 0.74 for Cho/Cr, indicating good classification performance. Validation results showed 75% accuracy, supporting their potential for clinical glioma grading. The optimal cut-off value trained from any two hospitals is validated at a third hospital, and the accuracies of Cho/Cr are 77.78%, 100%, and 65%, respectively.

**Conclusion:**

Multicenter data analysis confirmed the good classification accuracy of Cho/NAA and Cho/Cr in glioma grading, providing a reliable basis for clinical treatment and prognosis planning. This aligns with findings from other studies.

**Clinical trial number:**

Not applicable.

**Supplementary Information:**

The online version contains supplementary material available at https://doi.org/10.1186/s12880-026-02640-5.

## Background

Glioma represents the most prevalent primary brain tumor within the central nervous system. It is regarded by the medical community as one of the most formidable malignant brain tumors due to its high morbidity, high recurrence, high mortality, and low cure rate [1, 2]. It represents over 80% of all primary brain malignancies and approximately 30% of all brain tumors [3, 4]. Gliomas are typically heterogeneous, exhibiting variations in metabolic characteristics across different brain regions and tissues [2, 5]. The 2016 edition of the World Health Organization (WHO) CNS guidelines employs a four-grade classification system for gliomas, based on histological features and biological behavior. Grades I and II are classified as Low-Grade Gliomas (LGG), while grades III and IV are classified as High-Grade Gliomas (HGG) [6]. As the grade of glioma increases, so too does the degree of malignancy and aggressiveness.

In clinical practice, Magnetic Resonance Imaging (MRI) is a frequently utilized imaging modality for the examination of brain diseases, including gliomas. In conjunction with contrast-enhanced scans, it can assist in delineating the boundaries of the tumor, assessing the integrity of the blood-brain barrier, and characterizing the vasculature within the tumor. These features make MRI an important tool for determining the morphological characteristics of tumors. However, in clinical practice, conventional MRI images mainly reflect the anatomical structure of brain tissue and lack quantitative concentrations information of metabolites [7].

Magnetic Resonance Spectroscopy (MRS) is a non-invasive, radiation-free technique that can provide information about metabolites in the lesion area by detecting various metabolites in the brain, including N-acetyl-aspartate (NAA), choline (Cho), creatine (Cr), and others. The metabolite ratio (e.g., Cho/NAA, Cho/Cr, etc.) can be employed to initially grade gliomas for clinical and disease diagnosis. The metabolite content of different human tissues is typically displayed in the form of a spectral peak integral area. This spectral peak integral area is positively correlated with the level of metabolite concentration, and is used to determine the metabolite information of the location of the lesion and to identify the most invasive portion of the tumor. Furthermore, the differentiation between tumor and normal tissues can be achieved through the analysis of changes in metabolite concentrations and ratios. This enables an accurate diagnosis and differential diagnosis of brain tumors, as well as the prediction of glioma histopathological grading [8–12].

In clinical practice, there are notable discrepancies in the available treatment options and prognosis for different grades of gliomas. Therefore, it is important for physicians to grade brain gliomas in a timely and accurate manner. This can assist in the formulation of individualized treatment plans and provide a crucial reference for the assessment of prognosis. Lin D et al. [13] highlighted that LGG and HGG are treated differently, and the prognosis varies according to the grade of the tumor.

However, there remains a lack of consensus regarding the most effective and reliable metabolite ratios for predicting the grade of gliomas in clinical practice. Moreover, the criteria for predicting glioma grading by spectra metabolite ratios are not entirely consistent across different medical institutions.

## Materials and methods

### Clinical data

The work is a retrospective study of single-voxel MRS of 3T scanners. 74 patients with MRS pathohistological diagnostic report were collected from the following three hospitals: First Affiliated Hospital of Xiamen University (43 patients, from November 2016 to June 2024), Second Hospital of Lanzhou University (23 patients, from August 2022 to May 2024), and Zhongshan Hospital of Fudan University (Xiamen Branch) (8 patients, from July 2020 to May 2023).

#### Patient selection

**Fig. 1:**
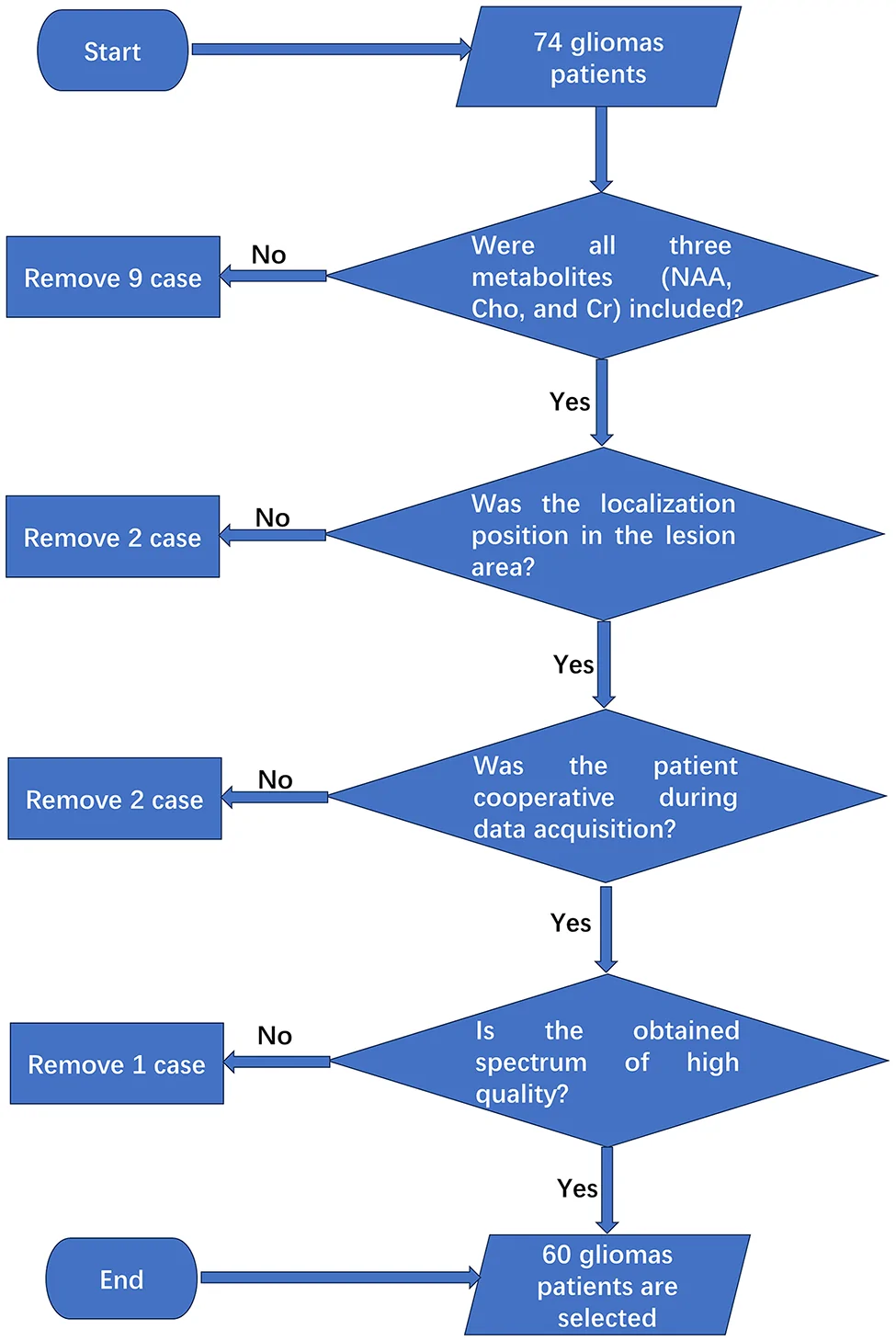
Flow chart of patient selection

Patients were selected according to the following criteria: (1) Were all three metabolites (NAA, Cho, and Cr) included in the absolute or relative values? (2) Was the localization position in the lesion area during wave spectrum acquisition? (3) Was the patient uncooperative during waveform acquisition? (4) Was the quality of the wave spectrum high? Figure 1 illustrates the process of data screening.

**Fig. 2:**
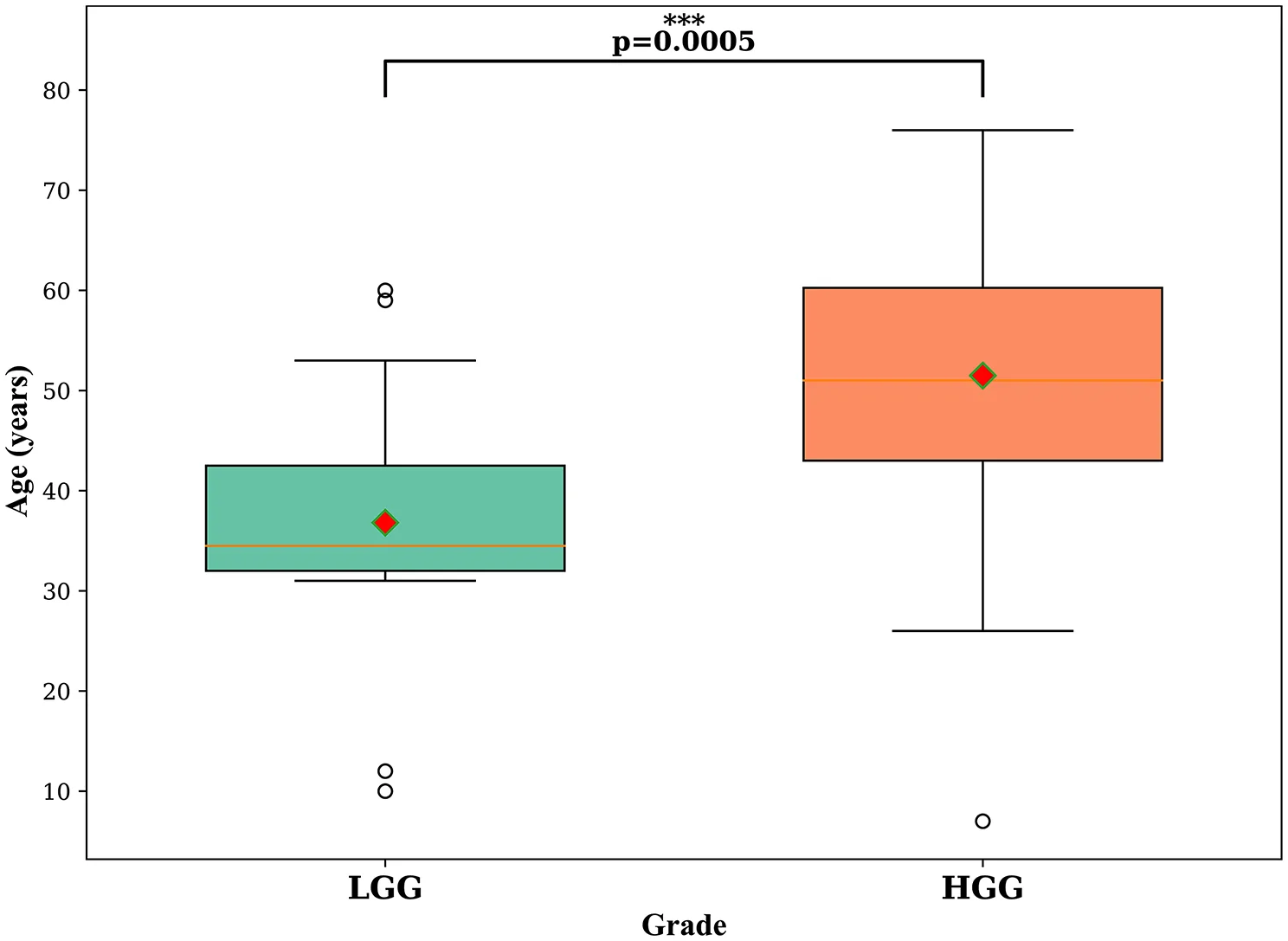
Age difference between LGG and HGG groups. There is a significant difference in the position of the upper and lower edges of the box line and the median line, and the *p* = 0.0005 (< 0.01) proves that the age difference between the two groups is statistically significant

The clinical data of the 60 patients were chosen in the ultimate analysis (Table 1). There are 44 patients with HGG (19 females, 25 males, 51.50 ± 13.56 years old) and 16 patients with LGG (7 females, 9 males, 36.81 ± 13.99 years old). The higher occurrence of HGG in older populations is expected. The distribution of tumor types within the LGG and HGG groups are as shown in Table 2. As Fig. 2 shows, the age difference between the two groups is statistically significant.

**Table 1:** Statistics of scanning device information for patients in the analysis

Time	Hospital	Scanner	Quantity	Total
2016.11.30-2024.06.19	The First Affiliated Hospital of Xiamen University	3T Siemens	31	36
		3T Philips	5	
2022.08.18-2024.05.06	The Second Hospital of Lanzhou University	3T Philips	20	20
2020.07.09-2023.05.04	Zhongshan Hospital of Fudan University (Xiamen Branch)	3T Philips	1	4
		3T GE MR750W	3	

**Table 2:** The distribution of tumor types within the LGG and HGG groups

Subgroup	G	O	A	other	Total
LGG	0	2	10	4	16
HGG	21	5	12	6	44

Note: **G**, **O** and **A** stand for glioblastoma, oligodendroglioma, and astrocytoma, respectively. **Other** 10 patients include 2 diffuse midline gliomas, 1 diffuse glioma, 1 NOS, and 6 NES

#### Index test

Four metabolite ratios were calculated: Cho/NAA, Cho/Cr, NAA/Cr, and NAA/(Cho + Cr). The objective of our study was to identify a spectral metabolite ratio that could more accurately differentiate between glioma grades and assess its validity in predicting glioma grades under multicenter conditions.

#### Reference standard

The histopathological diagnostic reports, which are considered the gold standard of brain gliomas. The 2016 edition of the WHO CNS guidelines employs a four-grade classification system for gliomas, based on histological features and biological behavior. Grades I and II are classified as LGG, while grades III and IV are classified as HGG.

#### Flow and timing

The 60 patients were all confirmed to have brain gliomas by histopathological diagnostic reports. All data were collected during preoperative examinations.

### MR spectroscopy protocols

All patient MR spectroscopy were conducted on 3T MRI scanners, including models from Siemens (MAGNETOM Verio 3.0T, Germany), Philips (Ingenia 3.0T and Ingenia 3.0T CX, Netherlands), and GE Medical Systems (Discovery MR750 3.0T, USA). These scanning plans were performed in accordance with the established clinical protocols at each center. MRS was conducted using a single-volume excited echo acquisition mode, with both short TEs (30ms and 35ms) and long TE (144ms), and a TR of 2000ms. The spectral volume of interest (VOI) is of a consistent size, 2.0 × 2.0 × 2.0 cm³. The voxel position is placed in the center of the tumor. The head coil has 32 channels. All 3T clinical scanners used in data collection employed conventional water suppress sequences for water suppress treatment.

**Fig. 3:**
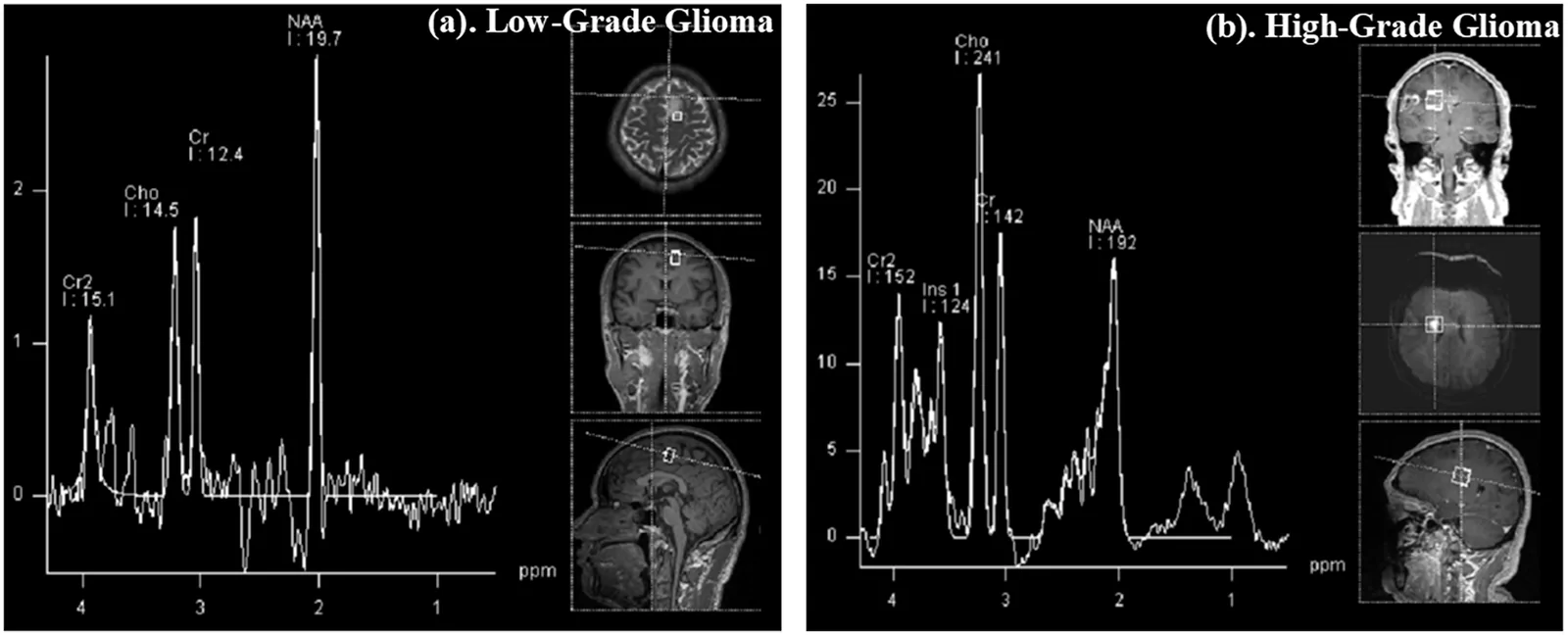
The actual spectra and the metabolite levels of distinct patients in single-pixel MRS. (**a**) is LGG, (**b**) is HGG. Note: These spectra were obtained via 3T Siemens scanning equipment at the First Affiliated Hospital of Xiamen University, and uploaded to the hospital PACS workstation for display

Zhongshan Hospital of Fudan University (Xiamen Branch) and Xiamen University Affiliated First Hospital store MRS data in the standardized DICOM (Digital Imaging and Communications in Medicine) format. with built-in vendor-specific quantitative algorithms capable of directly extracting concentrations of common metabolites such as NAA, Cho, and Cr. The concentrations of metabolites were calculated based on the spectral data of intracranial brain tumors, as well as the integral area under the spectral peaks. The following metabolite ratios were calculated: Cho/NAA, Cho/Cr, NAA/Cr, and NAA/(Cho + Cr). As Fig. 3 shows, the spectral characteristics of HGG are different from that of LGG, with a significantly increased Cr, a significantly decreased NAA, and both the Cho/NAA and Cho/Cr ratios were significantly increased.

**Fig. 4:**
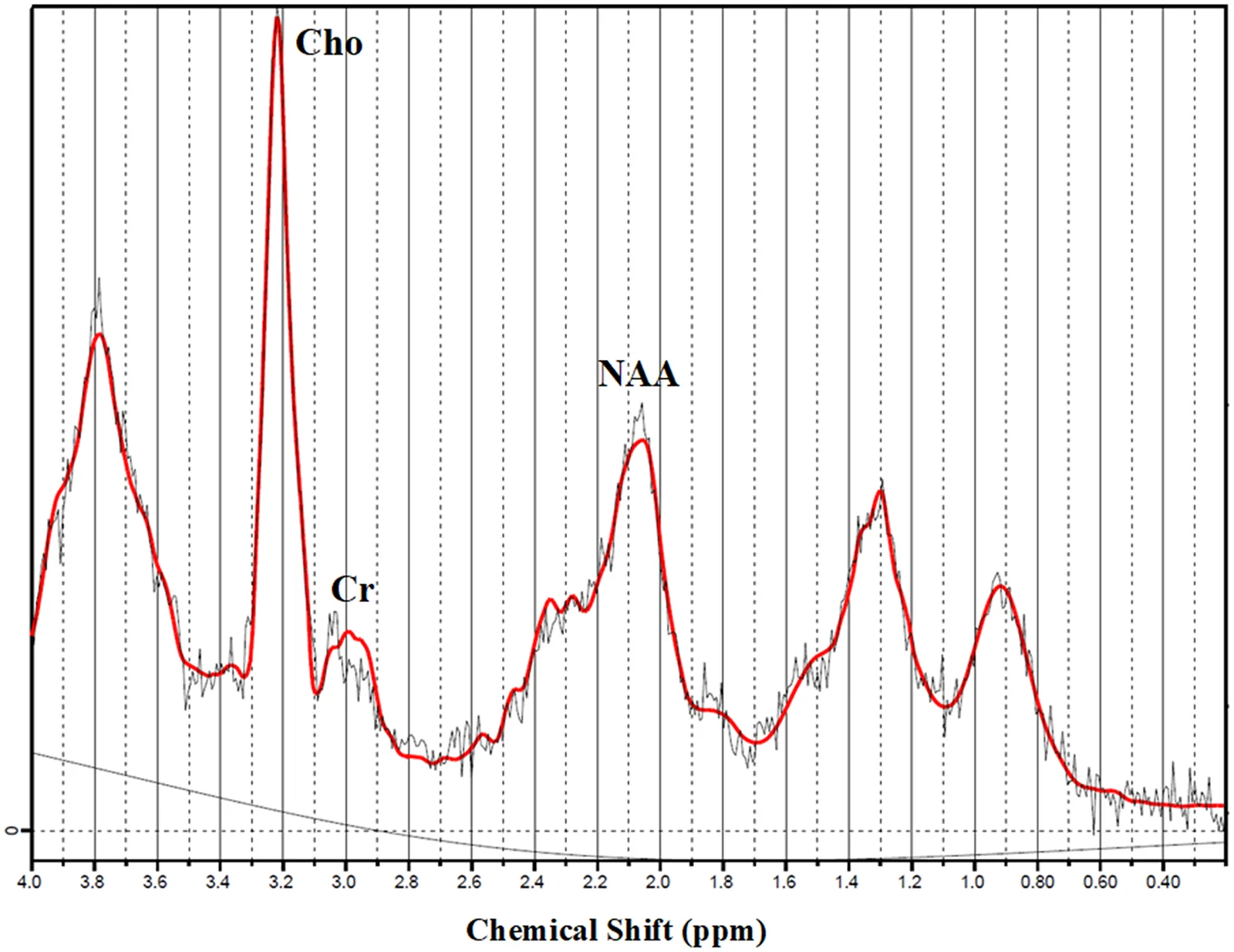
Magnetic resonance human metabolite spectra. Note: The spectra (black line) are real patient spectra acquired on a Philips Ingenia 3.0T CX scanning device at the Second Hospital of Lanzhou University on May 2023, and quantified (red line) by the CloudBrain-MRS platform of the Computational Sensing Group at Xiamen University

MRS data from Lanzhou University Second Hospital are in Raw Data format (raw k-space data), which require quantitative processing through the Intelligent Cloud Brain Imaging Magnetic Resonance Spectroscopy Quantitative Analysis Platform developed by the Computational Sensing Group at Xiamen University [14]. The quantification method used was the LC Model [15–19], where the relative concentration of Cho, NAA, and Cr are quantified with the relative concentration of total choline (tCho), total NAA (tNAA), and total Creatine (tCr), respectively. As Fig. 4 shows, NAA exhibits a chemical shift of 2.02 ppm, Cr displays a chemical shift of 3.03 ppm, and Cho demonstrates a chemical shift of 3.20 ppm. The MRS metabolite Cho/NAA ratio increases in a manner that is positively correlated with the tumor grade [3, 20–25].

### Analysis methods

In this work, the Shapiro-Wilk test (S-W test) [26] was initially employed to ascertain the normality of the four metabolite ratios for LGG and HGG. When both sample the *P*-value [27, 28] exceeded 0.05, the independent samples *t*-test (*t*-test) [29] were utilized, and vice versa, the Mann-Whitney U-test (U-test) [30] were employed.

Then, area under the curve (AUC) is calculated by plotting the Receiver Operating Characteristic (ROC) curves. The ROC curve is a graphical tool employed in the assessment of classification model performance [31]. The AUC of the ROC curve indicates the classification performance of the model at different cut-off values [32], which is used to assess the ability of the ratio to classify gliomas. An AUC below 0.5 indicates no classification ability, 0.5 represents random guessing, 0.5 to 0.7 shows low performance, 0.7 to 0.9 indicates good performance, 0.9 to 1 reflects excellent performance, and 1 signifies a perfect diagnosis.

**Fig. 5:**
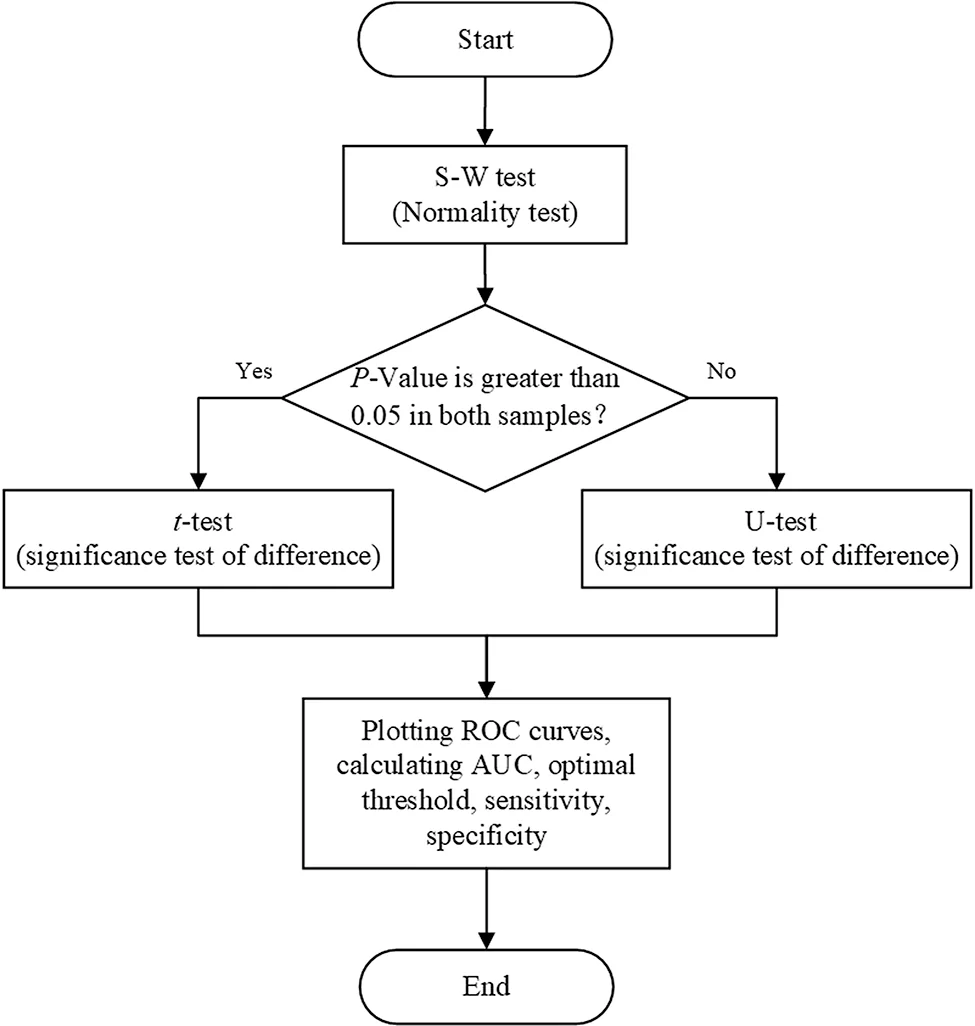
The overall analysis flowchart

Finally, the Youden index was employed to ascertain the optimal cut-off value for differentiating between LGG and HGG, that is, to identify the point at which the balance of sensitivity and specificity is optimal [33, 34]. The Youden Index values range from 0 to 1, with a value closer to 1 indicating a greater classification ability [35]. Figure 5 illustrates the analytical process.

The fundamental data pertaining to patients and associated information regarding MRS metabolites were classified, analyzed, and meticulously organized using Microsoft Excel 2021 software. The calculations and analyses were conducted using the Python Integrated Development Environment (PyCharm Professional 2023.1.2) [36]. The demographic data of the patients were subjected to analysis using the IBM SPSS Statistics 22.0 software [37]. All statistical analyses are subjected to rigorous scrutiny to guarantee the precision and reliability of the resulting data.

## Results

### Statistical analysis

First, all patient data were randomly divided into 80% (48 cases) for the analysis set, and 20% (12 cases) for the validation set. The analysis set comprised 48 patients, of whom 13 had LGGs (6 females and 7 males, 34.92 ± 14.23 years) and 35 had HGGs (15 females and 20 males, 49.71 ± 14.01 years). The validation set comprised 12 patients, of whom 3 had LGGs (1 female and 2 males, 45.00 ± 11.36 years), and 9 had HGGs (4 females and 5 males, 58.44 ± 9.23 years).

As Table 3 shows, in either the analysis set or the validation set, no significant differences were observed between the gliomas grade and gender. Therefore, the influence of gender on the grade of gliomas can be excluded.

**Table 3:** Age and gender information for patients with gliomas

	Group	Age (x̅±s)	
Analysis set (*n* = 48)	21 Females	45.52 ± 13.71	***P*** **= 1.00 > 0.05**
	27 Males	45.95 ± 17.73	
	13 LGG	34.92 ± 14.23	
	35 HGG	49.71 ± 14.01	
Validation set (*n* = 12)	5 Females	53.43 ± 13.38	***P*** **= 1.00 > 0.05**
	7 Males	57.40 ± 7.54	
	3 LGG	45.00 ± 11.36	
	9 HGG	58.44 ± 9.23	

Note: *P*-value indicates whether there is a significant difference between gender and the glioma grade, and *P* > 0.05 can be taken to mean that gender is not related to the glioma grade

Next, for the analysis set, the normality of the 4 metabolite ratios (Cho/NAA, Cho/Cr, NAA/Cr, and NAA/(Cho + Cr)) was verified of LGGs and HGGs using the S-W test. It is typically assumed that if a metabolite ratio satisfies a *P*-value of greater than 0.05 in both LGG and HGG, the ratio can be considered to conform to a normal distribution. The *P*-values of the S-W test for the 4 metabolite ratios are in Table 4.

**Table 4:** Analysis set of metabolite normality tests in glioma patients

Ratio	Grade	Statistic	*P*-Value
NAA/Cr	LGG HGG	0.858 0.939	0.036 0.052
Cho/NAA	LGG HGG	0.924 0.480	0.287 0.000
Cho/Cr	LGG HGG	0.748 0.897	0.002 0.003
**NAA/(Cho + Cr)**	**LGG** **HGG**	0.748 0.966	**0.758** **0.333**

Note: Metabolite ratios that obeyed a normal distribution (*P* > 0.05) at both LGG and HGG have been bolded. **Statistic** is a measure of whether the data adheres to a normal distribution between 0 and 1, with a value closer to 1 indicating a greater degree of conformity to a normal distribution

Figures A1-A4 in the Appendix show the normal distribution graphs of each metabolite ratio for the LGG and HGG groups. Figure A5 in the Appendix shows there was no significant correlation between all MRS markers and age in either LGG or HGG groups.

The results of the S-W test and the histogram of normal distribution indicated that the *P*-values of NAA/(Cho + Cr) were both greater than 0.05, thereby conforming to normal distribution, and the *t*-test was used. In contrast, the *P*-values of NAA/Cr, Cho/NAA, and Cho/Cr were less than 0.05 in at least one of the LGGs and HGGs and did not conform to normal distribution, and U-tests were employed. Table 5 presents the results of the *t*-test and U-test and Cohen’s d for the analysis set. From the Cohen’s d values, the difference between LGG and HGG, Cho/Cr > Cho/NAA > 0.5 > NAA/(Cho + Cr) > NAA/Cr.

**Table 5:** Metabolite ratios in the analysis set between the LGG and HGG groups

Ratio	LGG (*n* = 13)	HGG (*n* = 35)	Cohen’s d	t-test	U-test
				*P*-Value	*P*-Value
NAA/Cr	1.17 ± 0.87	1.21 ± 0.78	0.056	0.864	0.799*
Cho/NAA	1.24 ± 0.60	2.40 ± 2.82	0.476	0.150	0.017*
Cho/Cr	1.27 ± 1.09	1.97 ± 1.10	0.641	0.055	0.012*
NAA/(Cho + Cr)	0.47 ± 0.21	0.40 ± 0.20	0.378	0.250*	0.347

Note: *P*-value < 0.05 have been bolded to indicate a significant difference. * Indicates the *P*-value used for that metabolite ratio. A Cohen’s d value less than 0.2 indicates that the difference between the two groups is relatively small, 0.5 indicates that the difference between the two groups is of some practical significance, and 0.8 or above indicates that the difference is very significant [38]

**Fig. 6:**
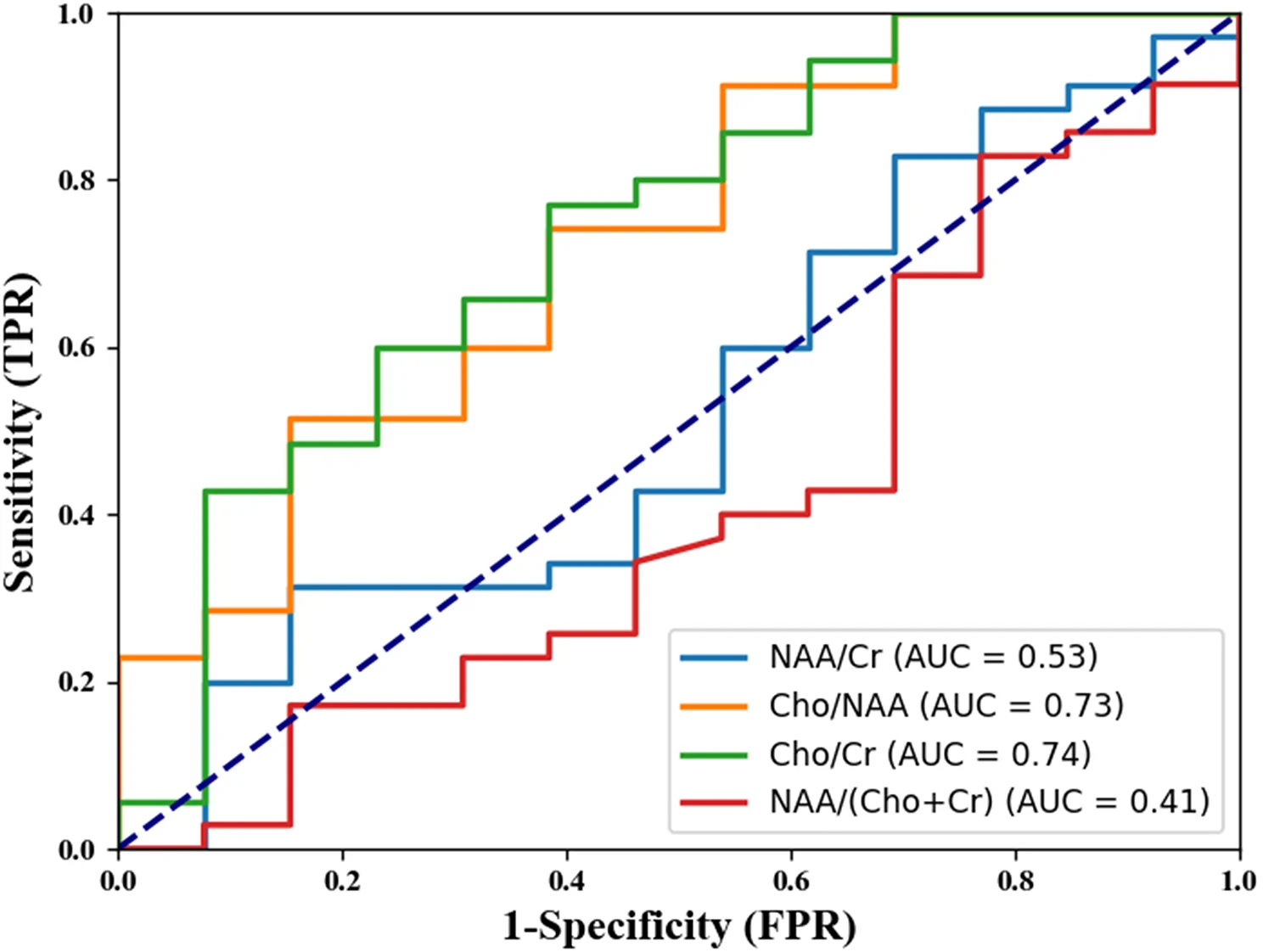
ROC curves for metabolite ratios of the analysis set (different ratios have been marked with different colors). Note: The sensitivity represents the proportion of correctly classified HGG, whereas the 1-specificity denotes the proportion of incorrectly classified HGG. The closer the ROC curve is to the upper left corner, the superior the classification performance

Then, using LGG as negative status and HGG as positive status, the classification performances of metabolite ratios were assessed by the area under the ROC curve at 95% confidence intervals. Figure 6 illustrates the ROC curves for the 4 metabolite ratios within the analysis set. The AUCs of the 4 metabolite ratios were as follows: NAA/Cr = 0.53, Cho/NAA = 0.73, Cho/Cr = 0.74, and NAA/(Cho + Cr) = 0.41. The AUCs of Cho/NAA and Cho/Cr were greater than 0.7, and the *P*-value were less than 0.05, indicating that Cho/NAA and Cho/Cr have superior classification performance in distinguishing LGG and HGG.

Finally, the effect of the optimal cut-off values of the 4 metabolite ratios on the grading of gliomas were assessed by calculating the Youden index. As Table 6 shows, for Cho/NAA, the optimal cut-off value was 1.011, the sensitivity was 91.43%, the specificity was 46.15%, and the *P*-value was 0.017 (*P* < 0.05); for Cho/Cr, the optimal cut-off value was 1.22, the sensitivity was 77.14%, the specificity was 61.54%, and the *P*-value was 0.012 (*P* < 0.05). In conclusion, the Cho/NAA and Cho/Cr ratios exhibited superior reliability and accuracy than other ratios in the grading of gliomas.

**Table 6:** Results of statistical analysis for the analysis set

Ratio	Cut-off Value	Sensitivity	Specificity	AUC	*P*-Value
NAA/Cr	1.602	31.43%	84.62%	0.53	0.799
**Cho/NAA**	**1.011**	**91.43%**	**46.15%**	**0.73**	**0.017**
**Cho/Cr**	**1.22**	**77.14%**	**61.54%**	**0.74**	**0.012**
NAA/(Cho + Cr)	0.234	82.86%	23.08%	0.41	0.250

Note: *P*-value < 0.05 and AUC > 0.7 have been bolded

### Accuracy verification

The accuracy of metabolite ratio classification is evaluated according to Eq. (1):

1 $$\:\begin{array}{c}Accuracy=\frac{TP+TN}{TP+FP+FN+TN}\end{array}$$

where, *TP* represents the number of HGG patients correctly classified as HGG, while *FP* denotes the number of HGG patients incorrectly identified as LGG. Similarly, *TN* signifies the number of LGG patients accurately designated as LGG, and *FN* represents the number of LGG patients misclassified as HGG.

To verify the accuracy of the two metabolite ratios, Cho/NAA and Cho/Cr, in grading brain gliomas, the validation set comprised 12 cases from 3 hospitals, representing 20% of the total number of cases included in the study.

We used the optimal cut-off value as a criterion to distinguish LGG from HGG. A metabolite ratio exceeding the optimal cut-off value indicates HGG, whereas a ratio below the optimal cut-off value indicates LGG. Specifically, Cho/NAA used 1.011 as the optimal cut-off value to grade 12 patients with gliomas in the validation set, and the results showed that the accuracy of grading using this criterion reached 75%. Similarly, Cho/Cr was validated using 1.22 as the optimal cut-off value and grading the same patients with the same 75% grading accuracy.

Besides, among the three hospitals, the optimal cut-off value trained by data from any two hospitals is validated at a third hospital (Table 7). For The First Affiliated Hospital of Xiamen University, the accuracies of Cho/Cr and Cho/NAA are 77.78% and 22.22%, respectively. For Zhongshan Hospital of Fudan University (Xiamen Branch), the accuracies of Cho/Cr and Cho/NAA are both 100%. For Second Hospital of Lanzhou University, the accuracies of Cho/Cr and Cho/NAA are 65% and 45%, respectively.

**Table 7:** Results on reproducibility across the different hospital centers

Hospital for validation	Metabolite ratio	*P* value	Cut-off value	Accuracy
The First Affiliated Hospital of Xiamen University	Cho/Cr	0.027	1.184	77.78%
	Cho/NAA	0.024	2.670	22.22%
	NAA/Cr	0.811	0.504	/
	NAA/(Cho + Cr)	0.085	0.234	/
Zhongshan Hospital of Fudan University (Xiamen Branch)	Cho/Cr	0.009	1.220	100%
	Cho/NAA	0.068	1.011	100%
	NAA/Cr	0.426	1.602	/
	NAA/(Cho + Cr)	0.724	0.234	/
Second Hospital of Lanzhou University	Cho/Cr	0.076	1.696	65%
	Cho/NAA	0.007	1.286	45%
	NAA/Cr	0.249	1.845	/
	NAA/(Cho + Cr)	0.027	1.830	/

## Discussion

These findings further support the potential validity of Cho/NAA and Cho/Cr for predicting brain glioma grading in the clinical setting. Peer related studies (Table 8) have similar findings [39].

**Table 8:** Previous MRS studies on gliomas grading

Metabolite	Task	Sample size	Equipment	Cross Center	Source
Cho/NAA (*P* < 0.05)	Grading (LGG/HGG)	35 patients	1.5T MRS (Siemens)	no	[7]
Cho/NAA (*P* < 0.05)	Grading (LGG/HGG)	71 patients	1.5T MRS (Toshiba)	no	[40]
Cho/tNAA (*P* < 0.001)	Grading (LGG/HGG)	26 patients	1.5T MRS (Siemens)	no	[41]
Cho/Cr(*P* < 0.01) MCM2 LI(*P* < 0.01) Cho/NAA(*P* = 0.023)	Grading (LGG/HGG)	43 patients	3T MRS (Siemens)	no	[39]
Glx/tCr (*P* < 0.005)	Grading (LGG/HGG)	26 patients	3T MRS (Siemens)	no	[42]
mI/Cr, Cho/Cr	Grading (LGG/HGG)	34 patients	1.5T MRS	no	[43]
Cho/Cr (*P* = 0.007) NAA/Cr(*P* = 0.009)	Grading (LGG/HGG)	25 patients	1.5T MRS	no	[44]
Cystat (*P* < 0.001)	Genotyping 1p/19q (codeletion/non-codeletion)	65 patients	3T MRS (Siemens)	2 hospitals	[45]
NAA/Cho(*P* < 0.01) Glx (*P* < 0.01)	Diagnosing (oligodendrogliomas/healthy)	15 patients	1.5T MRSI (Siemens)	2 hospitals	[46]

However, the majority of related studies are limited to single-center studies, which is relatively limited in scope and makes it challenging to comprehensively assess the diagnostic effects in different clinical scenarios. Lin [45] reported Cystat as a biomarker for 1p/19q Codeleted Gliomas cross 2 hospitals, and Glx levels can be used to assist in distinguishing low grade oligodendrogliomas from low-grade astrocytomas [46].

The increase in Cho and Cho/NAA ratio in MRS is positively correlated with tumor grade [47]. For in *vivo* ^1^H MRS applications, the NAA resonance has been used as a marker of neuronal density, which is supported by the observation of a decrease in NAA intensity in disorders which are accompanied with neuronal loss, such as tumors [47]. Choline-containing compounds (tCho) are involved in pathways of phospholipid synthesis and degradation, thereby reflecting membrane turnover. Increased choline signal is observed in cancer [48]. The concentration of total creatine (tCr) is relatively constant, with no changes reported with age [49] or a variety of diseases. As such the resonance of tCr is frequently used as an internal concentration reference [48]. Thus, Cho/NAA is better marker than NAA/(Cho + Cr).

Despite yielding clinically significant results, our study has notable limitations. (1) The sample sizes of LGG and HGG are different, which may have an impact on the generalizability of the findings. (2) Discrepancies may arise between the localization of lesions during MRS examinations and the sites of intraoperative pathological sampling. Furthermore, during the significant time interval between surgery and MRS examination, the tumor may have evolved from LGG to HGG. These factors may result in errors in grading. (3) The patient data was collected from multiple centers. Despite the standardization of data, potential discrepancies may arise due to variations in the equipment used for data collection, slice quality, the operators who conducted the collection, and the acquisition parameters employed. Furthermore, discrepancies between sample groups and inconsistencies in scanning equipment across different centers may impact the precision of metabolite ratios.

Future work includes validating the clinical applicability of these metabolite ratios in a multicenter setting with larger data volumes. Besides, it is crucial to standardize the acquisition and analysis of MRS data to minimize potential errors resulting from differences in equipment, operators, and acquisition parameters. These challenges not only facilitate a comprehensive assessment of the potential of MRS in glioma grading, but also ensure its clinical value and generalizability. We can consider other promising MRS biomarkers not included in this study. The amino acid Gln is an important component of intermediary metabolism and is primarily located in astroglia at a concentration of 2–4 mM [50]. At lower fields, the separation between Glu and Gln becomes unreliable, although the sum (often referred to as Glx) can be quantified with high accuracy [48]. mI is readily detected in short-TE ^1^H NMR spectra of the brain due to its high concentration of 4–8 mM [51], which has been proposed as a glial marker [52].

## Conclusions

This work systematically evaluated the classification performance of both the Cho/NAA and Cho/Cr metabolite ratios in 3T single-voxel for glioma grading under multicenter conditions, with the objective of validating their clinical applicability. The work analyzed diagnostic data from 60 patients with gliomas from three partner hospitals, with a particular focus on four metabolite ratios (NAA/Cr, Cho/NAA, Cho/Cr and NAA/(Cho + Cr)). 80% of the data were subjected to analysis, with the remaining 20% employed for validations. The results of the study demonstrated statistically significant differences (*P* < 0.05) between LGG and HGG for Cho/NAA and Cho/Cr. For Cho/NAA and Cho/Cr, the AUCs for were 0.73 and 0.74, respectively; the optimal cut-off values were 1.011 and 1.22, respectively; the sensitivities were 91.43% and 77.14%, respectively; and the specificities were 46.15% and 61.54%, respectively. The validation grading accuracy was 75% for both ratios. The optimal cut-off value trained from any two hospitals is validated at a third hospital. For the First Affiliated Hospital of Xiamen University, the accuracies of Cho/Cr and Cho/NAA are 77.78% and 22.22%, respectively. For Zhongshan Hospital, Fudan University (Xiamen Branch), the accuracies of Cho/Cr and Cho/NAA are both 100%, respectively. For Second Hospital of Lanzhou University, the accuracies of Cho/Cr and Cho/NAA are 65% and 45%, respectively. These findings further confirm the clinical utility of spectral metabolites.

## Supplementary Information

Below is the link to the electronic supplementary material.

Supplementary Material 1

## Data Availability

The datasets analyzed during the current study are not publicly available due to the privacy of the patients involved.

## Abbreviations

MRS: Magnetic Resonance Spectroscopy
NAA: N-acetylaspartate
Cho: Choline
Cr: Creatine
WHO: World Health Organization
LGG: Low-grade gliomas
HGG: High-grade gliomas
MRI: Magnetic Resonance Imaging
VOI: Volume Of Interest
S-W test: Shapiro-Wilk test
t-test: independent samples t-test
U-test: Mann-Whitney U-test
AUC: Area under the curve
ROC: Receiver Operating Characteristic
TPR: True-positive rate
FPR: False-positive rate
TP: True-positive
FP: False-positive
TN: True-negative
FN: False-negative
