# Supplementary material for: Gliomas grading prediction using multicenter 3T Magnetic Resonance Spectroscopy
Source: BMC Med Imaging. 2026 Jul 31;26:559. doi: 10.1186/s12880-026-02640-5 (PMC13715249; doi:10.1186/s12880-026-02640-5)
Supplement: Supplementary file 1 [file 12880_2026_2640_MOESM1_ESM.docx]

**Appendix**

Figure A1 to A4 illustrate the normal distribution of the four metabolite ratios in low-grade and high-grade gliomas. The horizontal axis represents the range of the data, while the vertical axis depicts the probability density of the data. Statistic is a measure of whether the data adheres to a normal distribution between 0 and 1, with a value closer to 1 indicating a greater degree of conformity to a normal distribution.

The blue line in the figure represents the kernel density estimation (KDE) curve, which depicts the actual probability density of the data. The black line, in contrast, depicts the theoretical normal distribution, which is based on the mean and standard deviation. A comparison of the two curves allows for the visualization of the discrepancy between the actual distribution of the data and the normal distribution. If the blue line and the black line are in close proximity or overlap, it can be inferred that the data align with the normal distribution. Conversely, a larger discrepancy between the two curves suggests that the data deviate from the normal distribution.

Figure A5 shows the correlation analysis between MRS markers and age. We can see that there was no significant correlation between all MRS markers and age in both LGG and HGG groups.

Figure A1. Normal distribution histogram of NAA/Cr of glioma. (a) LGG, and (b) HGG.

Figure A2. Normal distribution histogram of Cho/NAA of glioma. (a) LGG, and (b) HGG.

Figure A3. Normal distribution histogram of Cho/Cr of glioma. (a) LGG, and (b) HGG.

Figure A4. Normal distribution histogram of NAA/(Cho+Cr) of glioma. (a) LGG, and (b) HGG.

Figure A5. MRS markers and age-related analysis
